# Visuomotor Behaviour in Amblyopia: Deficits and Compensatory Adaptations

**DOI:** 10.1155/2019/6817839

**Published:** 2019-06-09

**Authors:** Ewa Niechwiej-Szwedo, Linda Colpa, Agnes M. F. Wong

**Affiliations:** ^1^Department of Kinesiology, University of Waterloo, Waterloo, Canada; ^2^Department of Ophthalmology and Vision Sciences, The Hospital for Sick Children, Toronto, Canada; ^3^University of Toronto, Toronto, Canada

## Abstract

Amblyopia is a neurodevelopmental visual disorder arising from decorrelated binocular experience during the critical periods of development. The hallmark of amblyopia is reduced visual acuity and impairment in binocular vision. The consequences of amblyopia on various sensory and perceptual functions have been studied extensively over the past 50 years. Historically, relatively fewer studies examined the impact of amblyopia on visuomotor behaviours; however, research in this area has flourished over the past 10 years. Therefore, the aim of this review paper is to provide a comprehensive review of current knowledge about the effects of amblyopia on eye movements, upper limb reaching and grasping movements, as well as balance and gait. Accumulating evidence indicates that amblyopia is associated with considerable deficits in visuomotor behaviour during amblyopic eye viewing, as well as adaptations in behaviour during binocular and fellow eye viewing in adults and children. Importantly, due to amblyopia heterogeneity, visuomotor development in children and motor skill performance in adults may be significantly influenced by the etiology and clinical features, such as visual acuity and stereoacuity. Studies with larger cohorts of children and adults are needed to disentangle the unique contribution of these clinical characteristics to the development and performance of visuomotor behaviours.

## 1. Introduction

Amblyopia is a common neurodevelopmental disorder clinically defined as reduced visual acuity that cannot be immediately corrected using optical refraction [1]. The current gold-standard treatment for amblyopia involves occlusion therapy; that is, patching the eye with better acuity to force the use of the amblyopic eye. However, monocular acuity deficits persist in 15-50% of children after treatment, and patients often have deficits in binocular visual function, such as reduced or absent stereopsis, interocular suppression, and gaze instability [2]. Importantly, in addition to the sensory visual deficits, there are a variety of changes in the perceptual, cognitive, and motor functions in children and adults with amblyopia (for reviews, see [3–5]).

The widespread effects of amblyopia on perceptual and sensorimotor functions are not surprising given that vision provides a key sensory input necessary for the optimal development of neural circuits and behavioural functions [3, 6–8]. Abnormal visual experience due to amblyopia during the sensitive periods of development has a direct effect on the morphology and neurophysiological responses of neurons in the striate and extrastriate cortex and the functional connectivity of cortical networks [9–14]. Because the primary visual cortex is the origin of two anatomically and functionally distinct neural pathways, i.e., the ventral stream that projects to the inferior temporal cortex and the dorsal stream that projects to the posterior parietal cortex [15], abnormalities in early visual processing can lead to profound changes in neural processing in cortical areas receiving inputs from the visual cortex [10, 16–18]. Support for the widespread cortical reorganization was recently shown in a study with 5 to 15-year-old children, which reported reduced functional connectivity density at rest in the anisometropic amblyopia group compared to an age- and gender-similar control group [19]. This study found decreased strength of both short- and long-range connections, which included pathways extending from the occipital cortex to the inferior temporal area, parietal cortex, and the prefrontal cortex. The authors suggested that the abnormal development of long-range connectivity could be the underlying neurophysiological cause of visuomotor deficits in amblyopia.

Visual perceptual changes in amblyopia have been studied extensively (for recent reviews, see [4, 20, 21]). In contrast, the effects of amblyopia on motor function have historically received relatively less attention, with most studies and new insights emerging in the last 10 years. Therefore, the purpose of this paper is to provide a comprehensive review of the literature that advanced our understanding of the neuroplastic changes in visuomotor behaviour in humans with amblyopia. To highlight the changes in motor function associated with decorrelated binocular visual experience during development, we begin by introducing the key components of visuomotor control.

## 2. Visuomotor Coupling during Goal-Directed Action

The only way to interact with our environment is through action: making eye movements while reading or looking for relevant objects, performing goal-directed reaching and grasping movements with our hands, or navigating to a target destination while avoiding obstacles. The intricate link between vision and movement was first revealed by Held and Hein [22] in a seminal study which demonstrated that normal development of functional vision requires specific experiences where visual and motor inputs are coupled during a particular behaviour. It is now widely accepted that vision provides a key sensory input during the performance of most daily activities, including reaching, grasping, and navigation [23–26].

Reaching and grasping is a complex behaviour, which requires spatial and temporal coordination among multiple sensory and motor systems. For example, following the decision to drink a cup of coffee, the action sequence requires coordinated movements of the eyes to fixate the cup, and then the arm to reach and grasp the cup without knocking it over or spilling its contents. This seemingly simple action requires extensive processing of the visual input along the dorsal cortical stream where neurons are preferentially activated by binocular inputs [27–30]. The first step requires localization of the object in three-dimensional (3D) space with respect to the body [31]. If the object falls on the peripheral retina, saccadic and/or vergence eye movements, as well as head movements, are executed to bring the image onto the fovea of both eyes. The fovea has the highest density of photoreceptors, and correspondingly, the largest representation in the primary visual cortex, which correlates with acuity thresholds [32]. In general, arm movements are initiated following object fixation [33–37], and this temporal coupling could reduce the sensory uncertainty about the object's extrinsic (i.e., 3D location) and intrinsic (i.e., material) properties. Reducing sensory uncertainty about an object's location and properties is important for planning efficient reach and grasp movements (i.e., feedforward control). Importantly, once the reach is initiated, online visual feedback provides information about the ongoing action, and can be used to amend the trajectory when errors are detected [38]. Prior experience and current visual information about an object's material properties are used to plan the grasp by predicting the amount of force necessary to grasp and lift the object [39, 40]. At the time of hand contact, haptic feedback becomes available and can be used to adjust the grip force when the initial visual information is not reliable [41, 42]. Establishing a stable grasp of the object is performed under visual control while fixating the object; however, as soon as the grasp is stable, the eyes move on to the next target [43].

As discussed above, vision thus provides important sensory input for optimal performance of goal-directed behaviours. When visual feedback is restricted or eliminated, movements become slower, less accurate, and more variable. For example, visuomotor performance is significantly diminished when adults with normal vision are forced to perform a task under monocular compared to binocular viewing [44–47]. Similarly, binocular vision provides an important sensory input for the performance of fine motor skills in typically developing children [48]. For example, lower stereoacuity thresholds (i.e., better stereoacuity) are associated with improved grasping performance in school-aged children [49]. On the other hand, children diagnosed with a developmental coordination disorder, which is characterized by reduced motor function, also exhibit abnormal binocular vision [50]. Given that the hallmark of amblyopia is a disruption in binocular visual function, it is important to understand the type and extent of neuroplastic changes in visuomotor behaviour in adults and children with this neurodevelopmental disorder. It is also important to note that neuroplastic changes can be negative, which would manifest as deficits, as well as positive, which would manifest as compensatory adaptations that allow individuals with amblyopia to function normally. Investigating the effects of amblyopia on the oculomotor, manual, and postural systems could provide insight into the neural adaptation of the brain networks involved in sensorimotor transformations underlying the performance of functional behaviours.

## 3. Profile of Amblyopia

Population studies in children estimate the prevalence of amblyopia to be between 1.3% and 3.6% [2]. Amblyopia arises when children experience decorrelated binocular visual input due to strabismus (i.e., eye misalignment), anisometropia (i.e., unequal refractive error), and strabismus and anisometropia (i.e., mixed mechanism) or form vision deprivation (e.g., infantile cataracts). In children younger than 3 years old, amblyopia is associated with strabismus in 82% of the cases, whereas in older children (3-7 years old), both anisometropia and strabismus each account for ~40% of amblyopia [2]. The clinical profile of visual deficits varies with the etiology of amblyopia. For example, strabismic amblyopia is associated with greater deficits in binocular visual function, including poorer stereoacuity and greater interocular suppression [51, 52]. A distinct pattern of visual deficits was also found among strabismic, anisometropic, and mixed mechanism amblyopia in a large cohort of adults [53]. Specifically, individuals with strabismus had reduced optotype acuity, as compared to grating acuity, and better than normal contrast sensitivity at low spatial frequencies. In contrast, adults with anisometropic amblyopia had comparable optotype and grating acuity but reduced contrast sensitivity. Because amblyopia etiology is associated with a different age of onset and differential effects on spatial vision, it is important to consider the role of amblyogenic factors on visuomotor development in children, and on motor performance in adults with amblyopia.

## 4. Effects of Amblyopia on the Oculomotor System

### 4.1. Fixation Stability

Periods of attempted fixation consist of fixational eye movements that include (slow drift velocity < 2 deg/sec), microsaccades, and high-frequency tremor [54]. The rate of microsaccades in visually normal adults is ~1-2 per second, and they tend to have a small amplitude, typically <0.5° with an asymptote at approximately 1°. The contribution of fixational eye movements to perceptual, cognitive, and motor functions is still under investigation. To date, studies with visually normal adults demonstrated that microsaccades prevent fading during a prolonged fixation (i.e., the Troxler effect) [55], they are associated with better performance during a high-acuity task (i.e., simulated needle threading) [56], and their rate increases during visual search [57]. These studies indicate that microsaccades play an important role in visual information processing. Consequently, abnormal fixational eye movements may contribute to deficits in performance of perceptual, cognitive, and motor tasks.

The effects of amblyopia on fixational eye movements have been studied for over 40 years, and several abnormalities have been documented in both adults and children with different amblyopia etiologies. The early studies included relatively small sample sizes, and they quantified fixation instability by reporting the frequency and amplitude of microsaccades, as well as amplitude and rate of ocular drift [58–61]. They showed that amblyopia etiology was associated with different patterns of fixation instability. Specifically, adults with strabismic amblyopia had an increased frequency of microsaccades (referred to as “saccadic intrusions” by the authors) in the amblyopic eyes, whereas individuals with anisometropic amblyopia had a comparable frequency of microsaccades to the control group [59]. In contrast, a higher amplitude and velocity of drift were evident in the amblyopic eyes regardless of etiology, but not in patients with intermittent strabismus without amblyopia [61]. Due to the small sample size, these studies could not disentangle the association between fixation instability and visual acuity.

Advancements in eye-tracking technology have led to larger studies in children and adults, providing insight into the association between fixation instability and visual acuity deficits. Recent studies quantified fixation instability using a variable called bivariate contour ellipse area (BCEA), which provides a measure of dispersion of the eyes during a fixation interval. Therefore, BCEA is a global measure of fixation instability influenced by the presence of microsaccades and ocular drift, so a larger BCEA could be due to an increased drift or more frequent and/or larger microsaccades. Results from two recent studies with adults are in agreement with the earlier reports. First, Gonzalez et al. [62] tested 13 patients with various amblyopia etiologies and found increased BCEA in the amblyopic eye, which was evident even during binocular viewing. In this small sample, amblyopic eye fixation instability was not associated with the etiology or visual acuity loss. In the second study, Chung et al. [63] assessed fixation instability in a larger cohort that included 14 adults with anisometropic and 14 adults with strabismic amblyopia. In agreement with the earlier studies [58, 59], they found a significantly higher rate and larger amplitude of microsaccades only in patients with strabismic amblyopia. Also consistent with previous studies [59], the amplitude of slow drift was greater during amblyopic eye viewing in all patients, regardless of amblyopia etiology. This was the first study in adults to show that microsaccade amplitude and slow drift amplitude are both associated with reduced visual acuity, and they explain 14% and 30% of total variance, respectively, in amblyopic eye acuity [63].

Several recent studies examined fixation instability in children with amblyopia. Subramanian et al. [64] assessed 52 children with amblyopia due to anisometropia, strabismus, or the mixed mechanism and compared their BCEA with those of nonamblyopic children (i.e., typically developing: *n* = 40 and nonamblyopic strabismus and anisometropia: *n* = 37). Fixation instability during amblyopic eye viewing was almost three times greater in comparison to fellow eye viewing in children with amblyopia, which in turn was comparable to the fixation stability found in the nonamblyopic children viewing with either eye and the left eye of a visually normal control group. This study did not report whether any of the children presented with latent nystagmus; however, fixation instability was larger in the horizontal axis, which is consistent with the presence of fusion maldevelopment nystagmus syndrome. Similarly to adults with amblyopia [63], amblyopic eye fixation instability in children was associated with poorer visual acuity (*r* = 0.60). Interestingly, this correlation was high in a subset of children with strabismic amblyopia (*r* = 0.94) but not statistically significant in the anisometropic amblyopia group (*r* = 0.26). Finally, although there was no significant difference in fixation instability among the subtypes of amblyopia, a trend towards significance was reported (*p* = 0.07). Several reasons could contribute to the lack of significant difference due to etiology in this study with children, which was clearly evident in the adult studies [58, 60]. First, the group with strabismic amblyopia consisted of only 7 children (6 with accommodative esotropia), whereas the anisometropic group included 21 children and the combined mechanism group included 24 children. Second, as mentioned previously, BCEA is a global measure of fixation instability influenced by microsaccades and drift. Based on the results from adult studies, it could be hypothesized that a larger BCEA in children with strabismic amblyopia is due to a larger microsaccade amplitude, whereas in children with anisometropic amblyopia the larger BCEA is due to larger drift amplitude.

Two additional studies provide further insight into the contribution of microsaccades and drift to fixation instability in the pediatric population. First, Shi et al. [65] tested 28 children with anisometropic amblyopia and compared their performance with an age-matched control group. They found a reduced rate of small-amplitude (i.e. <0.6 deg) microsaccades and an increased rate of larger-amplitude microsaccades during amblyopic eye viewing in comparison to fellow eye viewing, and when compared to children in the control group. These larger microsaccades also had significantly greater amplitude and peak velocity. The association with visual acuity was not examined in that study, presumably because all children had relatively mild amblyopia (range 20/30-20/60). The second study included 36 children with amblyopia and 11 visually normal children; however, 17 children were excluded from the analysis because they presented with latent nystagmus or unreliable eye-tracking data [66]. Therefore, the final sample included 19 children with amblyopia (9 with anisometropia, 4 with strabismus, and 6 with the mixed mechanism), and the severity of amblyopia ranged from mild (20/30) to severe (20/400). Results were in agreement with Shi et al. [65] and showed decreased frequency but increased microsaccade amplitude during amblyopic eye viewing. This behaviour was associated with amblyopia severity: microsaccade amplitude was approximately twice as large in children with severe compared to those with mild amblyopia. Surprisingly, there was no association with amblyopia etiology, which may be due to a relatively small sample size that included only 4 children with strabismic amblyopia. Interestingly, drift variance was higher in both the amblyopic and fellow eyes of children with amblyopia compared to the control group. Furthermore, the extent of drift variance was not associated with microsaccade amplitude or amblyopia severity in this particular study. It is difficult to directly compare these results with adult studies because Shi et al. reported the variance of composite eye position during the intersaccadic interval rather than the amplitude or velocity of slow drift.

The increased drift variance in both eyes of children with amblyopia might have implications for binocular oculomotor control, which was investigated by Kelly et al. [67]. They examined fixation instability of the amblyopic (nonpreferred) and fellow (preferred) eyes, as well as vergence instability during binocular viewing in a large cohort of children (amblyopic: *n* = 98 (49 anisometropic, 15 strabismic, and 34 mixed mechanism); nonamblyopic: *n* = 62  (15 anisometropic, 29 strabismic, and 18 mixed mechanism), and control: *n* = 46). Increased fixation instability (i.e., larger BCEA) was found in the amblyopic and nonamblyopic groups compared to the control group when viewing with the amblyopic or nonpreferred eyes. Moreover, vergence instability was associated with the presence of strabismus in both the amblyopic and nonamblyopic children. Further analysis showed that fixation and vergence instability were both associated with worse stereoacuity (*r* = 0.31). These results indicate that fixation instability is not a unique feature of amblyopia as children with different nonamblyopic visual and oculomotor deficits have significant fixation instability as compared to a visually normal control group.

To summarize, amblyopic eye fixation instability has been found in adults and children. Notably, most studies found no significant difference in BCEA between the control eyes and patients viewing with the fellow eye [62–65]. Comparing the results of studies with children and adults with amblyopia can reveal important insights about developmental plasticity. In particular, three studies with children found no significant association between etiology and fixation instability during monocular viewing [64–66]. In contrast, adults with strabismic amblyopia tend to exhibit significantly larger instability, which is mainly due to the increased frequency and amplitude of microsaccades [63]. One interpretation for these results could be that children with anisometropic amblyopia are able to improve their fixation stability during the course of development. In contrast, the presence of strabismus interferes with the normal development of oculomotor control. Therefore, the potential for positive plasticity may depend on the etiology of amblyopia.

Fixation instability has been associated with poorer visual acuity [63, 64, 66] and worse stereoacuity [61, 64], and these changes could have a negative impact on visuomotor behaviours, such as reading or visual search. This hypothesis was examined by two recent studies. First, Kelly et al. reported a moderate correlation between fellow eye fixation instability (i.e., BCEA) during binocular viewing and binocular reading speed (*r* = −0.52) in a cohort of 20 children with anisometropic amblyopia [68]. In contrast, the association between binocular reading speed and amblyopic eye fixation instability or stereoacuity did not reach significance (*r* = −0.32 and *r* = 0.22, respectively) in that study. Importantly, results from that study showed that children with anisometropia without amblyopia were reading at a comparable speed to a visually normal control group. Unfortunately, the fixation instability measures were not reported for that group. The second study by Chen et al. examined microsaccades during a visual search task in 21 children with amblyopia while viewing was monocular with either the amblyopic or fellow eye; however, binocular viewing was not assessed [69]. Visual search was significantly less accurate and longer during amblyopic eye viewing when compared to the control group. Although search accuracy was comparable between the groups when viewing with the fellow eye, search time remained significantly longer in children with amblyopia. Deficits were further exacerbated in children with latent nystagmus. Collectively, emerging studies suggest that fixation instability may influence functional tasks such as reading and visual search in individuals with amblyopia; however, more studies are needed to assess the role of etiology, visual acuity, and stereoacuity in the performance of these complex tasks.

### 4.2. Saccades

Voluntary saccades are quick, conjugate eye movements performed to explore the environment in a task-dependent manner [70]. Saccade metrics and their underlying neural networks have been studied extensively, and the subcortical and cortical areas involved in saccade planning and generation are well established [71–75]. At the behavioural level, saccades can be described by the “main sequence,” which quantifies the relation between saccade amplitude and peak velocity and amplitude and duration [76]. Saccades are often studied by asking participants to fixate a target presented in the periphery. The two outcome measures commonly used to assess oculomotor processing are saccade latency (i.e., reaction time) and amplitude, which provide a measure of a decision-making process, specifically, the time needed to detect and initiate a response towards a peripheral target and the accuracy and precision of target localization [77, 78].

The effects of amblyopia on saccadic eye movements were first characterized by Schor [79] in a small sample of five adults with strabismic amblyopia. Using a stimulus that stepped predictably along the horizontal meridian, results showed more variable saccade latency when viewing with the amblyopic eye; however, there was no significant difference in mean latency between the two eyes. The lack of difference was most likely due to the predictable nature of stimulus presentation because a subsequent study reported significant delays in saccade initiation for the amblyopic eye when a stimulus was presented with temporal and spatial uncertainty [80].

Building on these pioneering studies, research into saccadic eye movements has flourished over the past 10 years. Recent studies assessed larger cohorts of patients, and showed that clinical characteristics, such as amblyopia etiology, acuity, and stereoacuity have a significant influence on saccade latency and kinematics. The effects of etiology were clearly demonstrated in a large cohort (*n* = 393) of adults with abnormal vision [81]. The interocular saccade latency difference was 40-80 ms in adults with strabismic and mixed mechanism amblyopia and 25 ms in adults with anisometropic amblyopia. Perdziak et al. [82, 83] reported comparable differences in interocular saccade latency in a smaller cohort of patients with strabismic (*n* = 10) and anisometropic amblyopia (*n* = 16). In contrast, the cohorts assessed by Niechwiej-Szwedo et al. showed a different pattern of results [84, 85]. The mean latency difference between the amblyopic and fellow eye was ~45 ms in both anisometropic and strabismic amblyopia groups. It is possible that the discrepancy between this and other studies is due to patient characteristics; for example, 4 out of 13 anisometropic patients (31% of the sample) had a severe acuity loss and negative stereopsis [85], whereas only 1 of the 16 patients tested by Perdziak et al. in 2014 lacked stereopsis. In general, patients with anisometropic amblyopia have better binocular vision compared to those with strabismic amblyopia; however, nonbinocular observers with anisometropia show a similar pattern of visual deficits to the strabismic group [53]. Thus, it is possible that the increased interocular latency difference in a cohort of anisometropic amblyopia assessed by Niechwiej-Szwedo et al. was due to a greater number of nonbinocular participants.

A detailed regression analysis of patient characteristics, including acuity, stereoacuity, and ocular deviation, on saccade latency was performed in a cohort of 55 adults with various amblyopia etiology (22 anisometropic, 18 strabismic, and 15 mixed mechanism) [86]. The study also included a group of patients with strabismus only (*n* = 14) to disentangle the effects of ocular deviation (tropia) and amblyopia. Multiple regression analysis revealed that visual acuity loss was the strongest predictor of amblyopic eye saccade latency delay, explaining 28% of the total variance. These results are consistent with the McKee et al. study [81], which found a correlation of 0.75 between interocular saccade latency difference and interocular acuity difference.

It is important to mention that subtle, but significant saccade latency deficits may also be present when viewing binocularly or monocularly with the fellow eye. For example, binocular compared to monocular viewing is associated with superior performance on various perceptual and motor tasks in visually normal participants (i.e., binocular summation) [87–90]. Consistent with this literature, a significant, albeit small (~20 ms), binocular advantage was found for saccade latency in the control group, but not in the anisometropic or strabismic groups, where a comparable saccade latency was found during fellow eye and binocular viewing [84, 85]. In general, saccade latency elicited by a sudden onset of a peripheral stimulus is comparable between patients' fellow eye and a monocularly viewing control group [81]. Superficially, these results may seem at odds with a recent study that reported significantly longer saccade latency in strabismic amblyopes viewing with the fellow eye when compared to a monocularly viewing control group [82]. This discrepancy can be explained by considering the experimental paradigms used to assess saccades. Perdziak et al. used the disappearance of a central stimulus as the “go” signal to initiate saccades, which is in contrast to the other studies that used a peripheral stimulus to elicit reflexive saccades. These results provide the first evidence to suggest that strabismic amblyopia may affect saccade initiation when viewing with the fellow eye.

It is well known that saccade latency is task dependent; for example, latency is shorter when the fixation target disappears 50-200 ms prior to the presentation of a peripheral target, which is referred to as the gap effect [77]. It has been suggested that the reduced saccade latency in gap trials is due to the reduced activity of fixation neurons in the superior colliculus [91]. Two recent studies examined the gap effect in adults with amblyopia [92, 93]. Results showed faster saccade latency for gap trials across all viewing conditions suggesting that disengagement of visual attention is not affected by amblyopia. Notably, the delays for saccade initiation during amblyopic eye viewing persisted during the gap trials. In other words, during amblyopic eye viewing saccades were initiated faster during gap trials compared to overlap trials, but the latency was still longer compared to fellow eye viewing.

The accuracy and precision of saccade amplitude are important measures of performance; however, only one study to date assessed these outcomes in a cohort of 55 adults with amblyopia [84–86]. The study found no significant differences in saccade accuracy (i.e., mean amplitude or gain) between the patient and control groups. In contrast, saccade endpoint precision was significantly reduced across all viewing conditions in the anisometropic group [85], and a strong trend towards significance (*p* = 0.06) was found in the strabismic amblyopia group [84]. A detailed regression analysis revealed that 25% of the total variance in saccade amplitude precision was explained by visual acuity loss [86]. Finally, the presence of amblyopia was associated with the increased frequency of secondary, corrective saccades. It is plausible that these secondary saccades represent a compensatory/adaptive mechanism to correct for the spatial error following the primary saccade.

Reduced saccade precision and an increased number of secondary saccades could impact reading, which is an important daily activity that requires accurate and precise control of eye movements. Several studies that examined reading in amblyopia reported significantly lower reading speeds in children and adults, even during binocular viewing. For example, the reading speed of adults with strabismic amblyopia during binocular viewing was ~67% of that found in an education-matched control group [94]. Reading deficits were associated with a greater frequency of saccades [94, 95]. Studies with children indicate that reading speed is reduced by ~25% in the amblyopic eye compared to the fellow eye, even in the case when the acuity recovers to normal [96]. Two other studies assessed reading during binocular viewing and reported an ~33% reduction in reading speed in the amblyopia group when compared to a visually normal control group as well as children with strabismus only or anisometropia without amblyopia [68, 97]. These studies stress that it is amblyopia, rather than other oculomotor or visual problems, that has a negative impact on reading speed. Importantly, using eye-tracking during the reading task revealed differences in oculomotor behaviour, including greater frequency of regressive or forward saccades [68, 97]. These changes in saccadic behaviour during reading are consistent with studies that reported reduced saccade precision and an increased number of secondary saccades in a single target task [84, 85].

In summary, accumulating evidence demonstrates a significant saccade-related deficit in the amblyopic eye, which is evident regardless of amblyopia etiology. The impairment manifests as a delay in movement initiation and reduced precision of target localization. Moreover, saccade deficits are associated with acuity loss, and may be greater in adults with strabismic and mixed amblyopia [81, 86]. These behavioural deficits could arise as a result of difficulties in processing the sensory information and/or planning the motor response. Several lines of evidence suggest that oculomotor deficits arise from delays in the processing of sensory input. First, studies using electroencephalography and magnetoencephalography showed delayed responses in the visual cortex during amblyopic eye viewing in comparison to fellow eye or binocular viewing [98, 99]. Second, Perdziak et al. used a computational model approach to show that increased latency in amblyopia is due to a slower accumulation of visual information [83, 100]. It has also been argued that fixation instability could contribute to increased saccade latency [81, 92, 101]; however, this hypothesis has not been directly tested in amblyopia. Finally, it is important to note that although saccade deficits are largely confined to the amblyopic eye, some individuals may also exhibit subtle deficits in saccade latency and precision during binocular or fellow eye viewing, which could have a negative impact on visuomotor behaviours, such as reading.

### 4.3. Smooth Pursuit

Smooth pursuit involves conjugate eye movements that stabilize the image of a moving target on the fovea. Cortical networks involved in smooth pursuit include areas in the parietal and frontal, as well as subcortical areas, with some overlap with the saccade network [72, 102]. While saccades are initiated to reduce the retinal position error, the initiation of pursuit movements requires an estimate of the target's velocity based on retinal input and transformation of that sensory input into a motor output (i.e., matching eye velocity). A common metric used to evaluate the accuracy of smooth pursuit is gain, which is calculated as the ratio of eye velocity to target velocity. A gain of one indicates accurate tracking; that is, the image of a moving target is stabilized close to the fovea. The gain is dependent on a target's velocity, with values approaching 0.9 for target velocities between 10 and 90 deg/sec in visually normal humans [103]. Deficits in smooth pursuit may be expected in individuals with amblyopia given that disruption of the central vision is the hallmark of amblyopia.

Von Noorden led the first investigation into the effects of strabismic amblyopia on smooth pursuit and reported lower pursuit velocity and increased saccade frequency (i.e., catch-up saccades) [104]. These initial findings were corroborated by subsequent studies [79, 105, 106], which shed more light on the critical variables that impact smooth pursuit performance in amblyopia. First, a study that included strabismic and anisometropic groups reported that individuals with strabismic amblyopia exhibited greater deficits in the amblyopic eye, including reduced pursuit velocity, increased frequency of catch-up saccades, and nasal-temporal gain asymmetry [105]. These deficits were most pronounced for small-amplitude targets (<2 deg), where tracking was accomplished mainly with saccades rather than pursuit. Smooth pursuit was evident for larger target amplitude (4-8 deg); however, the gain was significantly lower than normal (0.4-0.7). Since individuals with strabismic amblyopia have increased fixation instability [58, 59, 63] with a strong bias for a nasalward drift [58], Bedell et al. assessed the influence of nasal drift on pursuit accuracy by subtracting the mean velocity of fixational drift from pursuit velocity [106]. Even after applying this correction for drift, results showed persistent reduction in pursuit gain for the amblyopic eyes. Overall, these studies provide clear evidence for smooth pursuit deficits in the amblyopic eye of strabismic amblyopes. In contrast, individuals with anisometropic amblyopia show relativity fewer deficits. Specifically, a recent study showed that the mean pursuit gain of both eyes was comparable to visually normal controls; however, pursuit initiation was slightly delayed (~20 ms), and the gain was more variable in the amblyopic eye [107].

### 4.4. Vergence

Vergence involves disjunctive eye movements to fixate objects presented at different viewing distances [108]. Disparity vergence is initiated during binocular viewing because objects nearer or farther from the fixation plane stimulate disparate retinal locations. Vergence eye movements are executed to reduce the disparity such that the images on both retinas fall on corresponding retinal points, and an object is perceived as single. Disparity vergence is neurally coupled with accommodative vergence, which is activated by visual blur [109]. Therefore, vergence can be initiated by the accommodative system during monocular viewing.

The effect of amblyopia on vergence eye movements was examined by Kenyon et al. [110] in seven adult amblyopes (4 strabismic, 3 anisometropic). In comparison to the control group, which showed symmetric vergence during binocular viewing, the strabismic group had asymmetric vergence eye movements, which were accompanied by saccades. Moreover, vergence dynamics were similar during binocular and monocular viewing, indicating a deficit in disparity-driven vergence, and the use of accommodative vergence when viewing binocularly. There was a lack of consistency in vergence behaviour in the anisometropic group: one patient performed similarly to controls, another patient's vergence was highly variable, and one performed similarly to the strabismic group. Clearly, further research with a larger sample size is necessary to gain a better understanding of the effects of clinical characteristics associated with amblyopia on vergence eye movements. Understanding the deficits and adaptation of the vergence system has ecological significance because everyday behaviours involve binocular eye movements. Furthermore, ocular vergence provides an important distance cue about the location of a fixated object, which is critical for planning and executing goal-directed reaching and grasping movements.

To summarize, deficits in eye movements are mainly seen during amblyopic eye viewing for saccades and smooth pursuit in adults with amblyopia. Given that the hallmark of amblyopia is an impairment in binocular vision, vergence movements are also affected; however, this should be examined in more detail with a larger cohort of patients. Importantly, eye movements have not been examined in children with amblyopia, which presents a significant gap in our understanding of how abnormal visual experience affects oculomotor development. Examining eye movements in children with amblyopia during the course of development will provide insight into the plasticity of the visuomotor system.

## 5. Effects of Amblyopia on the Manual System

Investigating upper limb movements provides insight into the neural control of visuomotor behaviour. For example, simple motor responses, such as a button press, have been used to assess the speed of sensorimotor processing, whereas reaching movements have been used to examine visuomotor mapping, motor planning (i.e., feedforward control), and feedback control [23, 31, 111–114]. One approach to study upper limb movement control involves using a high-speed motion camera to assess three-dimensional (3D) kinematics, including limb trajectory, velocity, and acceleration. In addition to measures such as movement latency, duration, and accuracy, which provide an overall index of motor performance, 3D kinematics provide insight into motor planning and feedback control processes. For example, reaction time, peak acceleration, and peak velocity have been used to assess feedforward control, while the duration of the deceleration phase and limb trajectory path have been used to infer feedback processes [115–119]. The aim of Section 5.1 is to synthesize the current knowledge about the effect of amblyopia on various components of visuomotor behaviour.

### 5.1. Stimulus Detection

Visuomotor control has been first studied in amblyopia using a manual reaction time paradigm to assess the speed of information processing during a simple stimulus detection task. Results showed increased reaction time for centrally presented targets during amblyopic compared to fellow eye viewing [120–123]. Notably, Hamasaki and Flynn reported a high correlation between visual acuity loss and reaction time in a cohort of strabismic amblyopes (*n* = 36; *r* = 0.82) [122]. Reduced contrast sensitivity in the amblyopic eye has been documented extensively [53, 124–126], and it is well known that reaction time is influenced by stimulus strength (Pieron's Law [127],). Therefore, several studies examined whether equating signal strength (i.e., contrast) across the two eyes reduces the latency delay in the amblyopic eye. These studies highlight important differences as a result of amblyopia etiology. Specifically, in the case of anisometropic amblyopia, there was no significant difference in the manual reaction time between the amblyopic and fellow eyes after stimulus visibility was equated [128]. On the other hand, individuals with strabismic amblyopia exhibited persistent delays during amblyopic eye viewing even when stimulus contrast was equated between the two eyes [101]. The authors suggested that this latency delay could be due to fixation instability, which is greater in adults with strabismic amblyopia [63].

Given that central vision deficits are the hallmark of amblyopia, delayed response initiation for targets presented centrally is not surprising. The manual reaction time to peripheral targets was subsequently assessed during monocular viewing by Chelazzi et al. in a group of people with amblyopia and esotropia which ranged from 6 to 40 PD [129]. Results showed longer manual latency when viewing with the amblyopic eye for stimuli presented in the central 10 deg as compared to more peripheral targets, which was interpreted as a stronger suppression of the central visual field in strabismic amblyopia. Manual button press responses to peripheral targets have not been examined in anisometropic amblyopia. However, Niechwiej-Szwedo et al. investigated manual pointing responses to high-contrast targets presented at 5 and 10 deg eccentricity [130]. Comparing the reaction time of the anisometropic amblyopia group with that of the control group showed no significant difference between the groups or viewing conditions. In contrast, reach initiation was significantly delayed in the strabismic amblyopia group compared to the control group, particularly for the amblyopic eye viewing condition [131], which is consistent with the results from Chelazzi et al. Regression analysis that included the full cohort (i.e., anisometropic, strabismic, and mixed mechanism groups) showed that visual acuity loss explained only 10% of the total variance in reach latency (compared to 28% of variance that was explained by reduced visual acuity for saccade latency in the same cohort) [86].

Altogether, these studies provide important insight into the effect of amblyopia on the speed of target detection during visuomotor processing. First, the delay in motor response initiation is longer in individuals with strabismic amblyopia compared to anisometropic amblyopia. Second, response initiation is delayed not only for centrally presented targets, but also for peripheral targets presented within 10 deg eccentricity. Third, poorer visual acuity is associated with increased manual response delay, but this relation appears to be stronger for centrally presented targets as compared to responses evoked by peripheral stimuli. Fourth, delays in response initiation persist in strabismic amblyopia after equating stimulus contrast between the two eyes. Overall, these results are consistent with studies that found increased saccade latency during amblyopic compared to fellow eye viewing. Importantly, the delay in both saccade and manual response initiation appears to be greater in individuals with strabismic compared to anisometropic amblyopia.

### 5.2. Spatial Localization

Abnormal space perception in humans with amblyopia has been documented using a variety of experimental tasks, such as stimulus bisection, alignment, drawing, or pointing [132–138]. Performance on these tasks provides information about the accuracy and precision of spatial localization, as well as spatial distortions. Despite the differences across experimental tasks used, results are remarkably consistent and indicate significant spatial localization deficits for the amblyopic eye, including systematic errors (i.e., lower accuracy) and increased uncertainty (i.e., reduced precision), as well as significant spatial distortions [136–139]. Although these spatial deficits seem to be most pronounced in central vision [140], some studies have reported abnormal spatial localization in the peripheral visual field by up to 15 deg eccentricity [141]. Interestingly, in the latter study, the distortions were highly heterogenous and not associated with clinical characteristics, such as visual acuity or strabismus. These results contrast with a study that examined spatial localization using an alignment task in children (*n* = 32) which showed that the group with strabismic amblyopia had larger constant and precision errors compared to the anisometropic and control groups [142]. Notably, reduced localization precision has been associated with poorer visual acuity in individuals with strabismic amblyopia (*r* > 0.80 for adults, *r* = 0.56 for children). Taken together, significantly greater perceptual spatial deficits have been reported for the amblyopic eye in strabismic compared to anisometropic amblyopia.

Most studies that examined spatial processing in amblyopia focused on perceptual alignment tasks, while only a few examined upper limb reaching/pointing responses. Given the dual visual processing streams [15, 143], it is important to examine the effects of amblyopia on spatial localization using both perceptual and motor tasks. Fronius and Sireteanu [134] examined pointing to targets presented briefly at 5-20 deg from fixation, with and without visual feedback of the arm in a cohort of 19 adults with amblyopia. They showed reduced accuracy and precision during amblyopic eye viewing in a group with strabismic amblyopia. In contrast, individuals with anisometropic amblyopia exhibited a relatively smaller increase in endpoint variability, and their overall performance was similar to the control group. Removing visual feedback of the arm was associated with increased endpoint variability, but this effect was similar across all groups. A more recent study also examined upper limb reaching movements in a large cohort of 55 adults with amblyopia during both monocular, as well as binocular viewing [86]. The main results showed reduced reach endpoint precision during amblyopic eye viewing as compared to monocular viewing in the control group and no differences during fellow eye or binocular viewing. Furthermore, a multivariate analysis revealed that amblyopic eye acuity and eye deviation accounted for 35% of the total variance in reach precision error.

In summary, two main findings emerge from studies that examined the effects of abnormal visual input during development on spatial processing. First, amblyopia is associated with spatial localization deficits across both perceptual (i.e., alignment) and motor (i.e., pointing) tasks. Second, spatial errors are greater in individuals with strabismic amblyopia. Two prominent models have been proposed to explain anomalous spatial processing in amblyopia. Hess et al. used the term “tarachopia” (i.e., scrambled vision) to describe the idea that neural representation of visual input from the amblyopic eye is distorted, which is also referred to as topographical disarray [125]. In contrast, Levi and Klein proposed that retinotopic undersampling of higher spatial frequencies could explain visual misperceptions [144]. These models were both developed to explain the perceptual deficits in spatial vision based on the results from stimulus alignment experiments. In order to explain the spatial deficits for a visuomotor pointing task, one must also consider the mapping of sensory input onto a motor response. In other words, the accurate and precise execution of upper limb movements requires the transformation of the sensory input, such as the spatial location of the target in egocentric coordinates, into an appropriate set of motor commands. Experimental evidence suggests that increased noise in the sensory signal representation due to topographical disarray or undersampling has a negative effect on the sensorimotor transformation process.

### 5.3. Feedforward and Feedback Control of Upper Limb Reaching and Grasping Movements

To gain a better understanding into how abnormal visual experience during development affects the control of upper limb movements, recent studies used kinematics to examine performance on reach-to-touch [130, 131, 145] and reach-to-grasp tasks [146–151]. The neural control of these movements is incredibly complex, and several theoretical models have been proposed in an attempt to explain the underlying mechanisms. Therefore, prior to considering the effects of amblyopia on upper limb movement performance, it is important to introduce a framework for sensorimotor control, and to define the kinematic outcome measures that provide insight into the control mechanism.

Optimal motor performance can be operationally defined as movements that are performed fast and accurately, while minimizing the energy and mental costs [152]. In order to perform the movement quickly, it is necessary to generate a large force (i.e., large impulse) to accelerate the limb towards the goal target. In general, increasing movement speed is associated with increased endpoint variability, which leads to a well-known speed-accuracy trade-off described by Fitts' Law [153, 154]. However, it is widely accepted that trajectory errors associated with greater limb acceleration can be amended during movement execution, provided that the movement duration is long enough and sensory feedback is available [119, 155]. Therefore, in the case of reaching movements, optimal motor performance depends on the interaction between feedforward control (i.e., generate a large force to accelerate the limb ballistically towards the target) and feedback control (i.e., use sensory feedback to correct trajectory errors as the arm approaches the target) [23, 156]. Recording limb kinematics using a high-speed motion camera provides insight into feedforward and feedback control processes [117, 157]. For example, peak acceleration occurs within the first 100 ms after movement onset. Therefore, it cannot be modulated based on sensory feedback and consequently reflects aspects of feedforward control (i.e., open-loop control). Typical reaching movements are longer than 500 ms; therefore, the latter part of the limb trajectory can be controlled using sensory feedback. Extensive evidence for online control comes from studies which show a significant reduction in spatial variability of limb trajectory after peak velocity [118, 155, 158].

Feedforward and feedback control processes have been examined in adults with amblyopia using kinematics for two different experimental tasks: reach-to-touch [159] and reach-to-grasp [148]. In addition, the developmental aspects of upper limb control have been assessed using a reach-to-grasp task in children with amblyopia [149, 151]. Reach-to-touch movements were studied by asking participants to point to a peripheral visual target randomly presented at 5 or 10 deg to the left or right of fixation as fast and accurately as possible. This relatively simple motor task revealed that amblyopia is associated with adaptation of feedforward control [130, 131]. This conclusion is supported by the following evidence. First, the overall movement duration was approximately 100 ms longer in the amblyopic group compared to the control group, regardless of viewing condition. Critically, partitioning the total movement duration into the acceleration and deceleration intervals (i.e., the time spent in the acceleration and deceleration phase), revealed a significant increase in the duration of the acceleration interval, while duration of the deceleration was not statistically different from the control group. In addition, the magnitude of peak acceleration was significantly lower in the amblyopic groups compared to the control group, which was evident across all viewing conditions. These results clearly show that amblyopia mainly affects the early movement kinematics, which reflect changes in feedforward control of upper limb reaching movements. Notably, changes in feedforward control (i.e., longer acceleration duration and lower peak acceleration) were associated with improved reach endpoint precision during binocular and fellow eye viewing, but not during amblyopic eye viewing. In contrast, the control group displayed a different control strategy in which a longer deceleration interval duration was associated with a higher endpoint precision. Results from the control group are consistent with a large body of research, which shows that optimal movement execution depends on the interaction between feedforward and feedback control where trajectory errors, due to a large initial acceleration force, are seamlessly corrected online to achieve fast, accurate, and precise movements [155, 157, 160, 161]. Importantly, artificially reducing visual acuity in one eye using plus lenses to simulate mild amblyopia in adults with normal vision did not affect their reach kinematics significantly [162]. Specifically, there were no changes in the feedforward or feedback control processes of reaching associated with a short-term, transient visual disruption in adults.

To summarize, detailed kinematic analysis revealed that decorrelated binocular visual experience during development is associated with a neural adaptation of the motor control system that involves an adjustment of the speed-accuracy trade-off function [163]. In other words, the available data suggest that in order to achieve similar movement precision to visually normal controls, individuals with amblyopia execute slower movements by reducing the initial acceleration (i.e., the ballistic part of the movement). Importantly, this adaptation in movement planning and execution allowed patients to achieve a similar endpoint precision during fellow eye and binocular viewing, but deficits persisted during amblyopic eye viewing. Additional regression analysis showed that more effective online control during amblyopic eye viewing was associated with better stereoacuity and smaller ocular deviation [86]. Finally, a similar visuomotor adaptation of reaching was evident regardless of amblyopia etiology, as well as in a group of adults with strabismus only, without amblyopia, which strongly indicates that normal binocular experience during development is necessary for optimal development of the visuomotor control system.

The planning and execution of reach-to-grasp movements is more complex compared to reach-to-touch because in addition to the transport component, grasping involves interacting with an object. In other words, in addition to localizing the target in egocentric coordinates to plan the reach movement [31], the central nervous system must process relevant object features to program grip aperture and grasp forces [39]. Grip aperture is defined as the distance between the thumb and index finger, and maximum aperture occurs around the time of peak deceleration [164]. Extensive research has shown that maximum grip aperture is scaled precisely to object size, such that a larger aperture is associated with larger objects. The fact that maximum grip aperture occurs during reach execution and that it is scaled to an object's size indicates that this kinematic variable is planned based on the initial visual input prior to reach initiation. Once the hand contacts the object, grip and load forces need to be generated to lift and transport the object. Studies have also shown that these forces are generated predictively based on an object's properties, such as weight, friction, density, and texture, which are encoded by the visual modality [113, 165–170]. Therefore, visual input provides critical sensory input for efficient performance of prehension movements.

Precision grasping has been studied in adults and children with amblyopia using a task that involves gripping cylindrical objects. The first study included a group of 20 adults with amblyopia (10 with strabismus and 10 with anisometropia; amblyopic eye acuity 0.20-2.80 logMAR) [148]. Movements performed during amblyopic eye viewing were slower, less accurate, and more inconsistent, and these deficits were apparent during transport and grasping. Although peak grip aperture was comparable between the groups during binocular and fellow eye viewing, impairment in grasp execution was evident once the hand contacted the object. Specifically, grasp application time was 22% longer, and grasping errors, defined as adjustments of the thumb and index finger around the object, were more than twice as high in the amblyopic group (control: 8.7% vs. amblyopia: 17.7%). In this study, visual acuity loss explained 50% of the total variance in grasp errors during binocular viewing. In contrast, etiology or stereo deficits were not significantly associated with prehension performance: grasping errors were similar in the groups with reduced and negative stereoacuity. A follow-up study used the same experimental approach to assess grasping in 20 individuals with a history of amblyopia, who had regained good visual acuity via occlusion therapy (i.e., 14 had normal acuity and 6 out of 20 had residual amblyopia with an acuity of 0.20-0.24) [150]. The results showed a similar pattern of prehension deficits during binocular viewing, with ~25% longer grasp application time and more than twice as many grasping errors. Consistent with Grant et al.'s study, no significant difference was found in grasp errors between the group with residual stereo and the stereo negative group. However, after removing a couple of outliers (2 out of 10), stereoacuity explained 63% of total variance in grasp application in the group with residual stereo. Overall, these studies provided the first insight into the effects of amblyopia on prehension performance using detailed kinematic measures. Both studies showed significant deficits during grasp application. Acuity and stereoacuity could be both contributing to these deficits; thus, a study with a larger sample size is required to disentangle their individual contributions.

Two other studies examined prehension in amblyopia while manipulating the environmental context. First, the effects of object contrast and lighting on prehension were examined in 13 adults with strabismic or mixed mechanism amblyopia [147]. The authors hypothesized that grasping deficits in amblyopia would be exacerbated when the task becomes more challenging; that is, when the object has low contrast or the task is performed in low lighting. Results from the study did not support this hypothesis: grasping performance was slower when the task became more challenging, but the relative changes were similar in both groups. In other words, the amblyopic group had a longer reaching and grasping duration even in the high-contrast and high-luminance condition, but when the task difficulty increased, they were not at a greater disadvantage in comparison to visually normal controls.

The second study examined prehension to objects surrounded by distractors/flankers in 20 adults with amblyopia. Using this experimental approach provides greater ecological insight into the effects of amblyopia on prehension because the objects that we interact with everyday are usually in proximity to other objects [146]. Results were consistent with previous studies showing slower overall performance, with a disproportionally greater deficit when the flanker objects were positioned in front or behind the target object as compared to either the left or right side. In contrast to the study by Grant et al., which found no significant difference in grip aperture between the groups during binocular viewing, aperture was significantly reduced in the presence of flanker objects in the amblyopic compared to the control group. These results indicate that patients with amblyopia adapted a more cautious approach strategy when reaching towards objects surrounded by flankers. It is possible that changes in prehension behaviour were due to visual crowding, which is one of the consequences of amblyopia [171]. On the other hand, changes in prehension could arise due to difficulties in estimating the depth of the target and flanker objects because the deficits were most pronounced when the flankers were presented in front or behind the object.

To summarize, significant prehension deficits have been found in adults with amblyopia during binocular viewing when interacting with high-contrast objects in a well-lit environment. In general, prehension was performed slower and the greatest impact was seen on grasp execution, rather than the reach component. The two measures commonly used to assess grasping are peak grip aperture and grasp duration. It is the latter measure that seems to be more impaired in amblyopia, which provides important insight into the nature of the control mechanism that is disrupted. First, results from Grant et al. [148] showed that peak grip aperture was scaled to object size and was comparable to the control group during binocular viewing. This suggests that despite having abnormal binocular input, individuals with amblyopia were able to extract the relevant visual information about the object's size to adjust their fingers appropriately during reaching. On the other hand, Buckley et al. [146] provided evidence to suggest that grip aperture was affected when the task became more challenging. Most importantly, all studies to date show significant grasping deficits after the hand contacts the object, which manifest as a prolonged handling duration. One interpretation for this finding is that patients have difficulty extracting, processing, or encoding specific object features that are critical for guiding the fingers' positioning around the object and/or for the programming of grip and lift forces. Because these forces are generated predictively based on the visual input acquired prior to contacting the object [39, 170], the specific pattern of grasping deficits in amblyopia indicates a compromised feedforward control of prehension. Prolonged grasping time while handling the object before the lift could be a compensatory strategy where haptic feedback plays a critical role in adjusting forces in order to grasp and lift the object successfully [172]. Several lines of evidence suggest that stereoacuity may play an important role in feedforward control of grasping forces [150]. First, grasping deficits persist in individuals with abnormal stereoacuity who “recovered” from amblyopia based on the current clinical definition (i.e., improved visual acuity). Second, reducing stereoacuity by simulating amblyopia with plus lenses leads to similar grasping deficits in visually normal individuals.

Results from adult studies are consistent with studies that examined prehension kinematics in a large cohort of 55 children with amblyopia aged 5 to 9 years old [149, 151]. Significant deficits, including longer movement duration and greater reach and grasp error rate, were found during binocular viewing in the younger group (5-6 yrs old), regardless of stereoacuity status. In contrast, stereoacuity was associated with improved grasping performance in the older group (7-9 yrs old). In fact, movement duration and grasping errors during binocular viewing were comparable in the control group and children with residual stereoacuity, but the group with negative stereopsis performed significantly worse. Most interestingly, a few longitudinal case studies presented in this paper showed that recovery of stereoacuity, but not just visual acuity, was associated with improved prehension kinematics. The results from this kinematic study suggest that children with better binocularity might be able to catch up to their peers, and with time develop appropriate sensorimotor strategies to perform similarly on this type of prehension task.

The fact that stereoacuity seems to be important for prehension and fine motor skills is also supported by a recent study that examined the effects of a newly developed binocular treatment (dichoptically presented iPod game) on fine motor skills in 18 children with mixed amblyopia etiology (mean age 8.5 yrs) [173]. Motor performance was assessed using a clinical test (the Bruininks-Oseretsky Test), which provides an overall, age-standardized score of motor proficiency. Following a 5-week training protocol, there was a significant improvement in stereoacuity (mean change 0.56 log arc sec) and an ~30% improvement in motor proficiency (mean change in standardized score 4.17). Interestingly, greater improvement in motor performance was associated with better baseline binocular vision (*r* = 0.75), rather than improvements in stereoacuity due to treatment. These results highlight the importance of binocular vision in the context of motor learning. Indeed, adults with poor stereovision show very little improvement after intensive training on a one-handed ball catching task [174].

Altogether, the accumulating evidence suggests that better binocular visual function, specifically stereoacuity, could provide a critical sensory input for the optimal development of prehension and other fine motor skills. Importantly, research shows that younger children seem to be affected to a greater extent compared to older children or adults. The improved performance of adults may be due to extensive practice and learning of compensatory strategies; for example, adaptation might involve relying on haptic feedback more when grasping and manipulating objects. However, studies with a larger sample size of adults and children over a larger age range (i.e., >9 yrs old) are required to establish a more definitive relation between stereoacuity and prehension performance, as well as the contribution of stereoacuity to motor learning. Using kinematics will provide useful insight into which aspects of sensorimotor control are affected, and how the system adapts to compensate for the abnormal visual experience during development.

## 6. Effects of Amblyopia on Balance and Gait

Maintaining postural stability while standing or navigating through the environment is of paramount importance for everyday function. Sensorimotor integration is key for postural stability control. Vision, along with vestibular and somatosensory inputs, provides sensory information about the position of the body in relation to the environment to ensure upright balance and forward progression during gait [175]. Postural stability during quiet stance is usually examined under increasingly challenging conditions, such as reduced base of support (i.e., standing on one leg) or reduced sensory cues (i.e., standing with eyes closed or on a soft surface). Similarly, obstacles have been used to assess walking in a more challenging environment [176].

There is a dearth of studies that examined the effect of amblyopia on balance and gait. Odenrick et al. provided the first report which included 23 children with strabismic amblyopia (the group also included 12 children with strabismus without amblyopia, aged 4.5-10.5 years) [177]. The results from the balance test showed that girls had significantly reduced postural stability, whereas boys performed similarly to the control group. Evaluation of gait parameters revealed that children with strabismus (and amblyopia) had significantly shorter stride length and shorter single-support time. This study found no association between binocular function and balance or gait measures.

The next study that examined the effect of impaired stereovision on adaptive gait included 16 adults (9 were amblyopic (5 had negative stereopsis), 7 were strabismic only (5 had negative stereopsis)) [178]. Gait parameters were assessed during an obstacle-crossing task where the task difficulty was manipulated using different obstacle heights, and the task was performed during binocular and monocular viewing. Detailed analysis of the gait pattern revealed that increasing the difficulty of the task by increasing obstacle height had a significantly greater impact in the stereo-deficient group that included individuals with amblyopia and strabismus. Specifically, increasing the obstacle's height was associated with slower gait velocity, a shorter step length when approaching the obstacle, and a higher toe clearance in the stereo-deficient group in comparison to the control group. Similar gait modifications were seen across all viewing conditions. The authors interpreted these results as a deficit in using visual input to plan the approach to the obstacle (i.e., feedforward regulation of the gait pattern). Other studies with visually normal subjects where binocular viewing was manipulated reached similar conclusions and suggested that monocular viewing disrupts the feedforward aspect of adaptive gait control [26, 179]. More specifically, increased uncertainty about an obstacle's height or location could lead to a more cautious approach that includes slower speed and higher toe clearance. Because the study did not report a separate analysis for subjects with and without amblyopia [178], it remains to be established whether amblyopia and strabismus have the same effect on adaptive gait, and the extent to which stereoacuity directly contributes to these changes in behaviour.

Finally, a recent study compared postural stability in a cohort of children with amblyopia (*n* = 18), strabismus without amblyopia (*n* = 16), and a visually normal control group (*n* = 22) [180]. Balance was assessed using the Bruininks-Oseretsky Test, and included standing tasks with increasing levels of difficulty; for example, standing in tandem or on one leg with eyes open or closed. The standardized balance score was significantly lower in the amblyopia group (mean 9.0) and the strabismic group (mean 8.6) in comparison to the control group (mean 18.9). Detailed analysis revealed the greatest deficits in the most challenging tasks; that is, when the base of support was narrow and visual input was removed in the eyes-closed condition. These findings suggest that despite abnormal vision, children rely on this sensory input to maintain balance and there is no evidence indicating compensatory adaptation involving the use of other sensory inputs (i.e., relying more on the vestibular or somatosensory input). Notably, there was no significant relation between balance scores and clinical patient characteristics, such as visual acuity or stereoacuity.

To summarize, evidence from a limited number of studies indicates that decorrelated binocular experience during development has a significant impact on the control of postural stability in children. Parallel research in adults with strabismus revealed reduced stability during quiet stance in comparison to a control group [181]. Surprisingly, the study showed better balance control when patients were viewing with the nondominant eye, and while performing a cognitive task. In contrast, another study with a larger cohort of children and adults with strabismus showed that postural stability was significantly worse only in children with strabismus, while adults performed similarly to a visually normal control group [182]. It appears that standing balance has not been examined in adults with amblyopia. This gap in knowledge should be addressed to clarify whether the deficits seen in children with amblyopia persist into adulthood or resolve at some age. Importantly, the deficits in balance control and gait are unmasked under more challenging testing conditions.

## 7. Conclusion

The goal of this paper was to provide a synthesis of current knowledge highlighting the changes associated with amblyopia across the three motor systems: oculomotor, manual, and postural. The accumulating body of research indicates that decorrelated visual experience during the early childhood years has a significant impact on visuomotor behaviour, including eye movements and upper limb reaching and grasping, as well as postural stability control. Examination of performance measures across different tasks shows that deficits are clearly evident during amblyopic eye viewing. These deficits include increased latency, slower execution, and reduced movement precision. Importantly, binocular viewing is also associated with some behavioural deficits, such as reduced reading speed, slower prehension, and decreased postural stability. In-depth kinematic analysis revealed that patients adapt compensatory strategies to improve performance. These compensatory behaviours involve secondary corrective eye movements, adjustment of the speed-accuracy trade-off function, and increased reliance on somatosensory feedback when manipulating objects. It is possible that the compensatory behaviours that are seen during binocular and fellow eye viewing depend on higher level cortical plasticity involving changes in connectivity and function of large cortical networks beyond the primary visual areas [19]. Importantly, using these compensatory strategies is associated with improved movement accuracy and precision; however, the cost is time: motor tasks are performed significantly slower. Moreover, it seems that deficits become more apparent when the tasks become more difficult or challenging, and that individuals with strabismic amblyopia may be affected to a greater degree. In regard to the sensorimotor control mechanism, experimental results suggest that amblyopia impacts feedforward and feedback movement control processes. Changes in feedforward control were most apparent during the performance of simple reaching movements, while feedforward and feedback control processes were both affected during prehension. Specifically, grasp execution was slower because the initial movement plan (i.e., feedforward control) was less accurate, which consequently led to a prolonged execution time in order to correct the errors. Finally, the majority of research examining the consequences of amblyopia on visuomotor function in humans focused on adult behaviour; therefore, our understanding of the developmental changes during childhood is quite limited. Addressing this gap in knowledge will provide important insights into the extent of neural plasticity and the clinical characteristics that influence positive and negative plasticity (i.e., compensatory adaptations and deficits).

### 7.1. Clinical Implications for Assessment and Treatment

Accumulating evidence indicates that binocularity, rather than just monocular visual acuity, is the critical sensory input contributing to optimal development of the sensorimotor control system. Correlated binocular experience during sensitive periods of development may be necessary for the normal development of the sensorimotor systems involved in the execution of eye movements, upper limb movements, and postural stability. Most intriguingly, developing innovative therapies that target the visuomotor system might facilitate the recovery of binocularity [183]. Emerging research highlights the functional impact of amblyopia on behaviours that involve spatiotemporal coordination among the visual and motor systems [3, 5, 184]. Yet, the effects of amblyopia on motor skill performance are not currently assessed during routine clinical assessments. Given the experimental evidence reviewed in this paper and additional studies that reported motor deficits on clinical tests [185–187], it may be important to consider adding a visuomotor assessment in this population to have a more comprehensive phenotype profile of individuals affected by amblyopia.
